# Genetic determinants of *Mycobacterium tuberculosis* adaptation and drug efficacy during stationary phase growth

**DOI:** 10.1128/spectrum.01096-25

**Published:** 2025-08-12

**Authors:** Xiao-Jie Shi, Kun-Xiong Shi, Fu Han, Li Wang, Xia Cai, Guo-Ping Zhao, Wei Sha, Liang-Dong Lyu

**Affiliations:** 1 Key Laboratory of Medical Molecular Virology of the Ministry of Education/Ministry of Health, Department of Medical Microbiology and Parasitology, School of Basic Medical Sciences, Fudan University 12478, Shanghai, People’s Republic of China; 2 CAS-Key Laboratory of Synthetic Biology, CAS Center for Excellence in Molecular Plant Sciences, Shanghai Institute of Plant Physiology and Ecology, Chinese Academy of Sciences 56690, Shanghai, China; 3 Shanghai Clinical Research Center for Tuberculosis, Shanghai Key Laboratory of Tuberculosis, Shanghai Pulmonary Hospital, Shanghai, People’s Republic of China; 4 Department of Microbiology and Microbial Engineering, School of Life Sciences, Fudan University 12478, Shanghai, China; ICON plc, London, United Kingdom

**Keywords:** *Mycobacterium tuberculosis*, nongrowing, drug resistance, drug tolerance, Tn-seq

## Abstract

The adaptation of *Mycobacterium tuberculosis* (Mtb) to a slowly growing or nongrowing state in growth-limited conditions plays a crucial role for drug tolerance. Although the mechanisms of Mtb adaptation under growth-limited conditions have been extensively studied, it remains unclear to what extent the cellular processes necessary to sustain nongrowing state affect drug efficacy. To investigate this, we performed a genome-wide transposon mutant screen, which allowed parallel identification of the genes that influence bacterial fitness and drug efficacy during the stationary phase. Our analysis revealed that genes encoding the SOS response, membrane phospholipid biosynthesis, proteasomal protein degradation, and cell wall remodeling critically determine Mtb fitness in both stationary-phase condition and antibiotic exposure. Surprisingly, we found that many mutants that compromise stationary-phase adaptation result in increased fitness during antibiotic treatment, including the recently identified genetic markers associated with poor clinical outcomes. Furthermore, genes involved in cell envelope biosynthesis and remodeling, antibiotic efflux, and phosphate transport are significantly enriched in the mutants sensitized to antibiotics, indicating that reduced drug entry is a critical factor that limits antibiotic efficacy in nonreplicating Mtb. We demonstrated that mutants deficient in utilization of lipids, the primary carbon sources for Mtb during infection, became tolerant to killing by rifampicin. We provided genetic and metabolic evidence that the activities of lipid metabolism are associated with rifampicin efficacy. These findings provide the detailed assessment of Mtb genes necessary for adaptation to the stationary phase and drug treatment and new insights into the mechanisms of antibiotic tolerance in nongrowing Mtb.

**IMPORTANCE:**

It has long been known that antibiotic efficacy is generally proportional to the bacterial growth rate. Yet it remains unclear how and to what extent the growth arrest-induced physiological and metabolic changes affect drug efficacy. Using the genome-wide transposon mutant screen, we identified the mutants that influence *Mycobacterium tuberculosis* adaptation and drug efficacy during the stationary phase of growth. We revealed both positive and negative correlations between stationary phase adaptation and drug sensitivity and identified many mutants that compromise stationary phase adaptation and result in increased fitness during antibiotic treatment, including the identified genetic markers associated with poor clinical outcomes. These results provide new insights into the mechanisms of antibiotic tolerance in nongrowing Mtb and suggest potential targets for drug development.

## INTRODUCTION

Tuberculosis caused by Mtb infection is one of the most devastating diseases of mankind (1). The standard chemotherapy for drug-susceptible tuberculosis consists of a 2-month induction phase with isoniazid (INH), rifampicin (Rif), pyrazinamide, and ethambutol, followed by a 4-month consolidation phase with at least rifampicin and isoniazid (2). The consolidation phase was found to be necessary to avoid relapse (2), although most patients no longer have culturable Mtb in their sputum after the first 2 months of therapy. Both the clinical and the animal model data suggest that the requirement of prolonged treatment is attributable to the presence of slowly growing or nongrowing drug-tolerant Mtb (3, 4).

The slowing of Mtb growth during infection was considered to result from the growth-limited conditions in the lesions (5, 6). In fact, various external cues, including the change from exponential growth to the stationary phase (7), nutrient deprivation (8), phosphate depletion (9), hypoxia (10–12), suboptimal pH (13), and reactive oxygen and reactive nitrogen species (6, 14), were found to greatly limit Mtb growth and reduce antibiotic efficacy. Notably, recent studies have demonstrated that Mtb clinical isolates exhibit diverse growth characteristics in host-like metabolic and drug conditions, and the slow growth phenotype may contribute to poor tuberculosis outcomes such as cavitary disease and treatment failure (15–17). For example, the clinically prevalent variations in glycerol kinase GlpK, propionate metabolism regulator PrpR, and Rv1339 (a cyclic nucleotide-degrading phosphodiesterase) were found to confer slow growth on glycerol and propionate and lead to multidrug tolerance.

It has long been known that antibiotic efficacy is generally proportional to the bacterial growth rate (18). Yet it remains unclear how and to what extent the growth arrest-induced physiological and metabolic changes affect drug efficacy. For example, while the Mtb mutants deficient in triacylglycerol synthase Tgs1 and isocitrate lyase Icl1 exhibited contrasting growth phenotypes under hypoxia, they were equally sensitized to antibiotic killing, suggesting that bacterial growth changes under growth-limited conditions may not be well correlated with antibiotic sensitivity (12, 19, 20). In this connection, recent studies have demonstrated that compared to the growth rate, the bacterial metabolic state more accurately correlates with antibiotic lethality (21, 22). Moreover, given that the anti-tuberculosis drugs exhibit distinct efficacy against nongrowing Mtb (3), the mechanisms underlying these drug-specific effects of bacterial growth arrest remain elusive.

In this study, we performed a genome-wide transposon mutant screen and identified the Mtb genes that influence bacterial adaptation and drug efficacy in the stationary phase, a typical model that is widely used for studying nongrowing states of bacteria in the laboratory (23). Our analysis provided a quantitative description of the responses of all single-gene mutations to stationary-phase growth inhibition and drug challenges. Our data revealed both positive and negative correlations between stationary phase adaptation and drug sensitivity, suggesting that disrupting the bacterial adaptive response to growth arrest may not uniformly disable bacterial antibiotic tolerance. Finally, through comparing the phenomic profiles of anti-tuberculosis drug Rif, streptomycin (Str), and ofloxacin (Ofx), we provide new insights into the mechanisms of antibiotic tolerance in nongrowing Mtb and demonstrate that the activity of lipid metabolism is correlated with rifampicin efficacy.

## RESULTS

### Transposon library construction and evaluation

**Fig 1:**
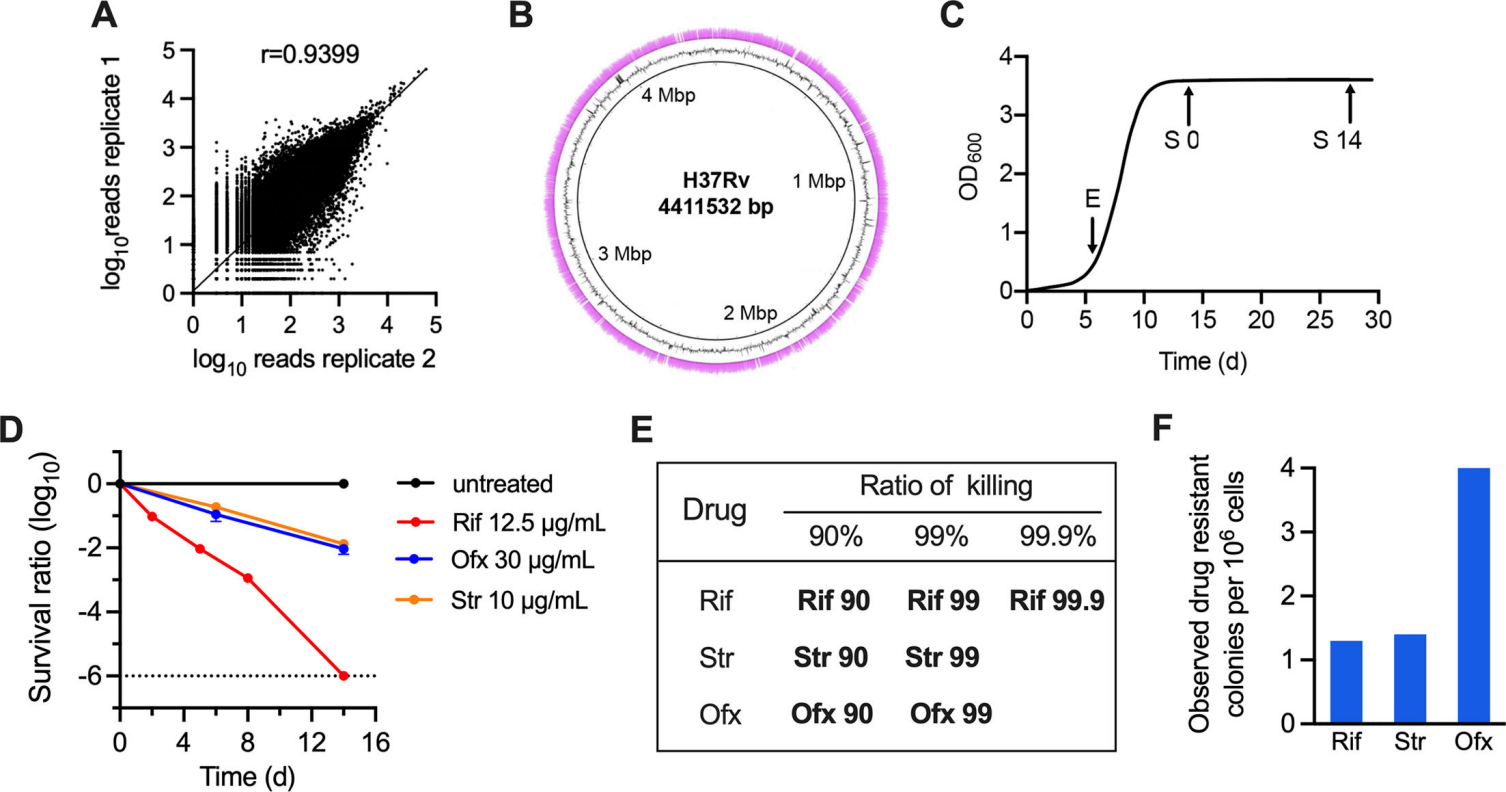
Quality control of the Tn-Seq and screening strategy. (**A**) Correlation coefficients of the number of reads per insertion site for two biological replicates of the input transposon library. Shown are log_10_(reads number +1). (**B**) Transposon insertion sites identified by Tn-seq. The number of reads corresponding to each insertion site is represented as pink bars mapped onto the H37Rv chromosome. Gray contour represents the GC content of the chromosome. Nucleotide positions are indicated. (**C**) Growth of the pooled library in 7H9OADC. E, exponential phase, S0 and S14, cultures that cease increasing optical density for 1d and 15d. (**D**) Survival of the pooled library exposed to antituberculosis drugs. Survival was determined by monitoring colony-forming units and expressed as the ratio compared with pre-treatment. Data are shown as the mean ± SE of two independent experiments for Tn-seq. (**E**) The samples used for Tn-seq were collected at the time points when antibiotic achieved 90%, 99%, and 99.9% killing of the bacterial population. (**F**) Spontaneous drug-resistant mutations in stationary phase Mtb exposed to indicated drugs.

A transposon insertion library containing approximately 200,000 independent mutants was generated in Mtb strain H37Rv using a modified himar1-based transposon (24). To assess the library diversity, the mutants were pooled in 7H9 media supplemented with OADC and allowed to grow into the exponential phase. Genomic DNA was extracted from the culture, randomly fragmented, and ligated to asymmetric adapters. The transposon-chromosome junctions were amplified by PCR and used for Illumina sequencing (Tn-seq) (25). The normalized read count of the two biological repeats (containing 7 to 13 million reads) exhibited a Pearson’s correlation coefficient of 0.94, demonstrating the high reproducibility of this methodology (Fig. 1A). We identified transposon insertions at 49,597 of the 74,605 TA sites in the H37Rv genome, corresponding to a density of 11.3 insertions per kilobase on average (Fig. 1B and Table S1). According to a prior estimation (26), this library covered 79% of the genomic TA sites that permit insertion. We next used the Hidden Markov Model (HMM) to predict gene essentiality under standard growth conditions (25, 26). Our analyses found 12.1% of Mtb genes are essential genes (483), 77.5% are non-essential genes (3,092), 4.9% are insertion growth defect genes (195), and 5.3% are insertion growth advantage genes (214) (Table S2). Of the 3,989 genes encoded by Mtb, 3,092 nonessential genes were identified by HMM analysis of the Tn-seq data (Table S2), which showed 90% consistency with the nonessential genes reported in a previous literature with the same growth condition (26).

### Selection of transposon mutations altering stationary phase adaptation and antibiotic efficacy

To estimate each mutant’s fitness during the stationary phase and antibiotic treatment, the pooled library was cultured in 7H9 media supplemented with OADC, allowed to grow into stationary phase (defined as a cease in increasing optical density for 1d [S0]) and then exposed to the first-line tuberculosis antibiotics Rif and Str, and the second-line drug Ofx at 100 × minimal inhibitory concentration (MIC) (Fig. 1C). These drugs inhibit DNA replication (Ofx), transcription (Rif), and translation (Str), which are the most representative cellular processes targeted by antibiotics. Our results showed that the cultures at S0 and S14 had similar colony-forming units, suggesting the bacterial population was in a nongrowing state (Fig. 1D, the untreated control). Consistent with previous observations (3, 7, 10, 14), our results showed that the nongrowing Mtb became highly refractory to the bactericidal action of the tested drugs (Fig. 1D). Rif, a cornerstone of modern antituberculosis therapy, retained its sterilizing activity against stationary-phase Mtb, whereas Str and Ofx could only achieve 99% killing at 14 d post-treatment (Fig. 1D). Approximately 1.5 × 10^6^ bacilli (10 times the library diversity) at various time points of treatment were collected, washed to avoid drug carryover, and recovered by plating (Fig. 1C and E), and then the genomic DNA was extracted from the pooled outgrowths for Tn-Seq analysis. The nonparametric resampling method was used to identify mutants that were differentially represented between conditions. For each gene, the normalized read counts at all the insertion sites under each condition were summed. The difference in the summed read counts between the two conditions was calculated and expressed as a log_2_ fold change (log_2_FC). Because the screen depends on the outgrowth, it should be noted that mutants with changed growth properties after removal of antibiotics, e.g., viable but not-culturable cells (5), will also be identified in this study.

A potential confounding factor that may influence our analysis is the emergence of spontaneous drug-resistant mutations during sampling. To assess this, we measured the drug-resistance mutation frequency in drug-exposed cultures and found that the probability of sampling a spontaneous drug-resistant mutant is very low (Fig. 1F). In addition, owing to the stochastic nature of mutational events, the potential epistatic effect of drug-resistant mutations in an individual insertion mutant could be eliminated by the statistical analysis of experimental replicates.

### Identification of genes required for Mtb adaptation to the stationary phase

**Fig 2:**
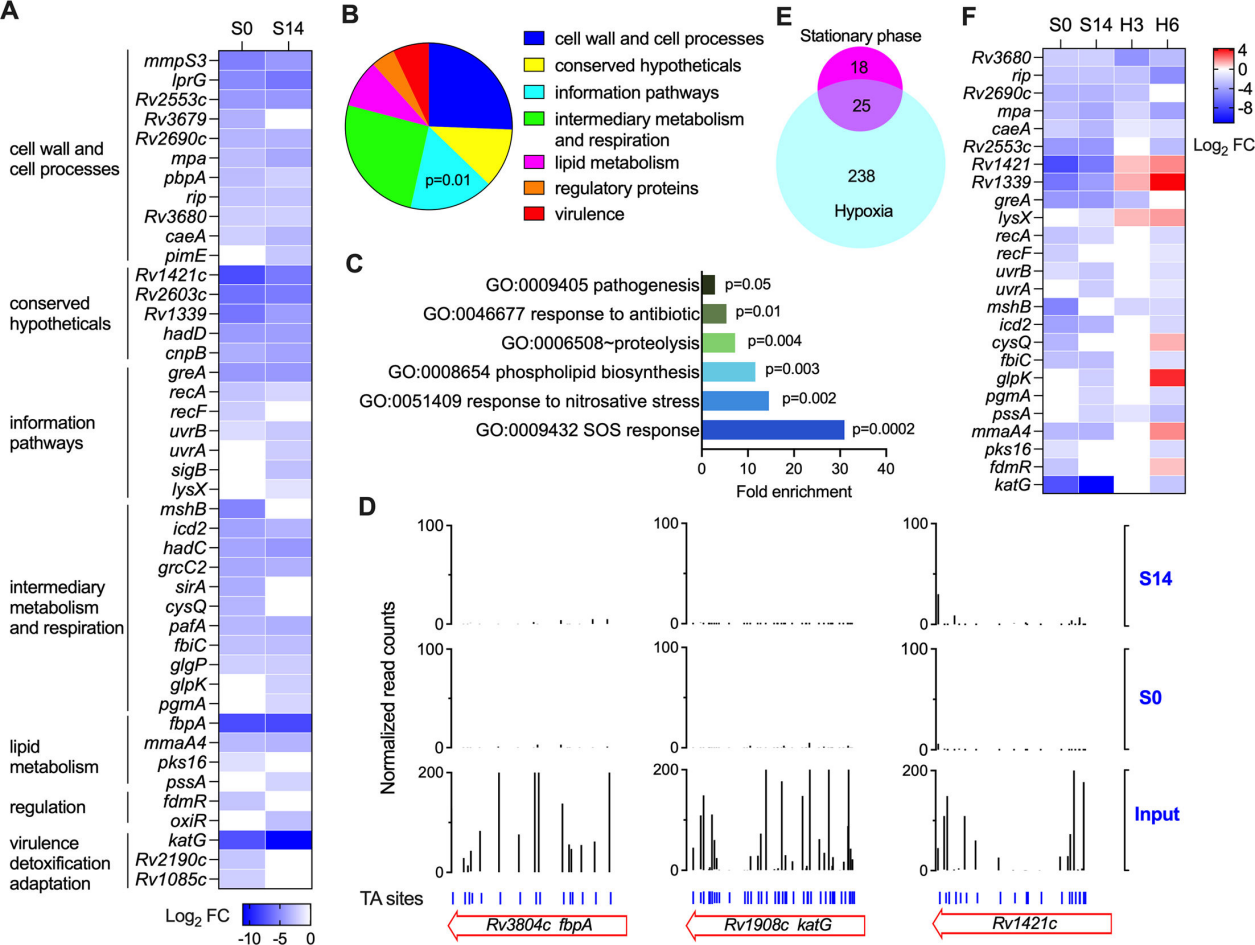
Genes required for Mtb adaptation to the stationary phase. (**A**) Heatmap showing the log_2_FC of the identified genes required for adaptation to the stationary phase. Data with non-significant change were shown as white cells. (**B–C**) Functional categories (**B**) and GO enrichment analyses (**C**) of the identified genes. (**D**) Comparison of transposon insertion abundances at different conditions (S0, S14, and input). (**E**) Venn diagrams showing the number of genes required for adaptation to the stationary phase and hypoxia conditions. (**F**) Heatmap showing the log_2_FC of the genes required for adaptation to the stationary phase and hypoxia conditions. H3 and H6, 3- and 6 weeks post-hypoxia. Data with nonsignificant changes were shown as white cells.

Genes that were specifically required for Mtb adaptation to the stationary phase were selected according to the two criteria: i) insertion mutations in these genes caused log_2_FC > 1 or log_2_FC < -1 with adjusted *P*-value < 0.05, and ii) should not cause growth defects in exponential phase growth. According to these criteria, a total of 43 genes were identified in this study (Fig. 2A and Table S3). All these mutants showed reduced fitness during the stationary phase (Fig. 2A). It should be noted that the fitness reduction may result from a reduced abundance in the stationary phase culture or a compromised ability to recover from the stationary phase.

According to TubercuList classification, functional categories related to information pathway were significantly enriched (Fig. 2B and Table S3). These genes mainly encode DNA repair proteins, including DNA recombinant repair factors RecA and RecF and nucleotide excision repair protein UvrAB, and transcriptional accessory factors such as the transcription fidelity factor GreA and sigma factor SigB. Gene Ontology (GO) analysis revealed that functional categories related to SOS response (*recA*, *recF*, and *uvrAB*), proteolysis (*mpa*, *pafA*, *caeA*, *rip*, and *recA*), phospholipid biosynthetic process (*pimE*, *lysX*, and pssA), and response to antibiotic (*katG*, *lprG*, *greA*, *lysX,* and *recA*) and nitrosative stress (*uvrB*, *fbiC*, *mpa*, and *pafA*) were significantly enriched among the identified mutants (Fig. 2C). Of note, several genes (*lysX*, *cysQ*, *uvrA*, and *caeA*) found to confer acid resistance were identified in our screen (27), which is consistent with the observation that stationary phase cells are associated with acid resistance (28).

The most marked fitness changes (log_2_FC < -7) were observed with the mutants with insertions in the *fbpA*, *katG*, and *rv1421c* (Fig. 2D). FbpA (also known as antigen 85A) is a component of the cell wall-synthetic enzymes (antigen 85 complex) responsible for the transfer of mycolic acids to α-α’-trehalose to form α-α’-trehalose monomycolate (TMM) and α-α’-trehalose dimycolate (TDM), also known as cord factor (29). A previous study found the *fbpA* knockout mutant grew similarly to the parent strain H37Rv in enriched media but exhibited no growth in nutrient-poor media and macrophages (30), which are consistent with our Tn-seq results. Mtb catalase KatG is a well-characterized drug-resistance target of INH and required for establishing persistent infection in mice (31). Although structural and sequence alignment analyses suggested a role of Rv1421 in peptidoglycan synthesis, its biological function in Mtb remained unclear (32).

Hypoxia was perceived as a frequently encountered growth-limiting host microenvironment during Mtb infection (10–12). Of the 43 genes required for adaptation to the stationary phase, 25 genes were found to contribute to Mtb adaptation to hypoxia (33) (Fig. 2E). Most of the shared genes showed reduced fitness in both conditions upon mutation, including those related to DNA repair, proteolysis, and antioxidation activities (Fig. 2F). Interestingly, there were seven genes (*rv1421*, *rv1339*, *lysX*, *cysQ*, *glpK*, *mmaA4*, and *fdmR*) exhibiting contrasting fitness change in the stationary phase and hypoxic conditions (Fig. 2F), suggesting the nongrowing Mtb cells induced by both conditions may have distinct physiological and metabolic characters.

### Mutants with altered antibiotic efficacy in stationary phase Mtb

**Fig 3:**
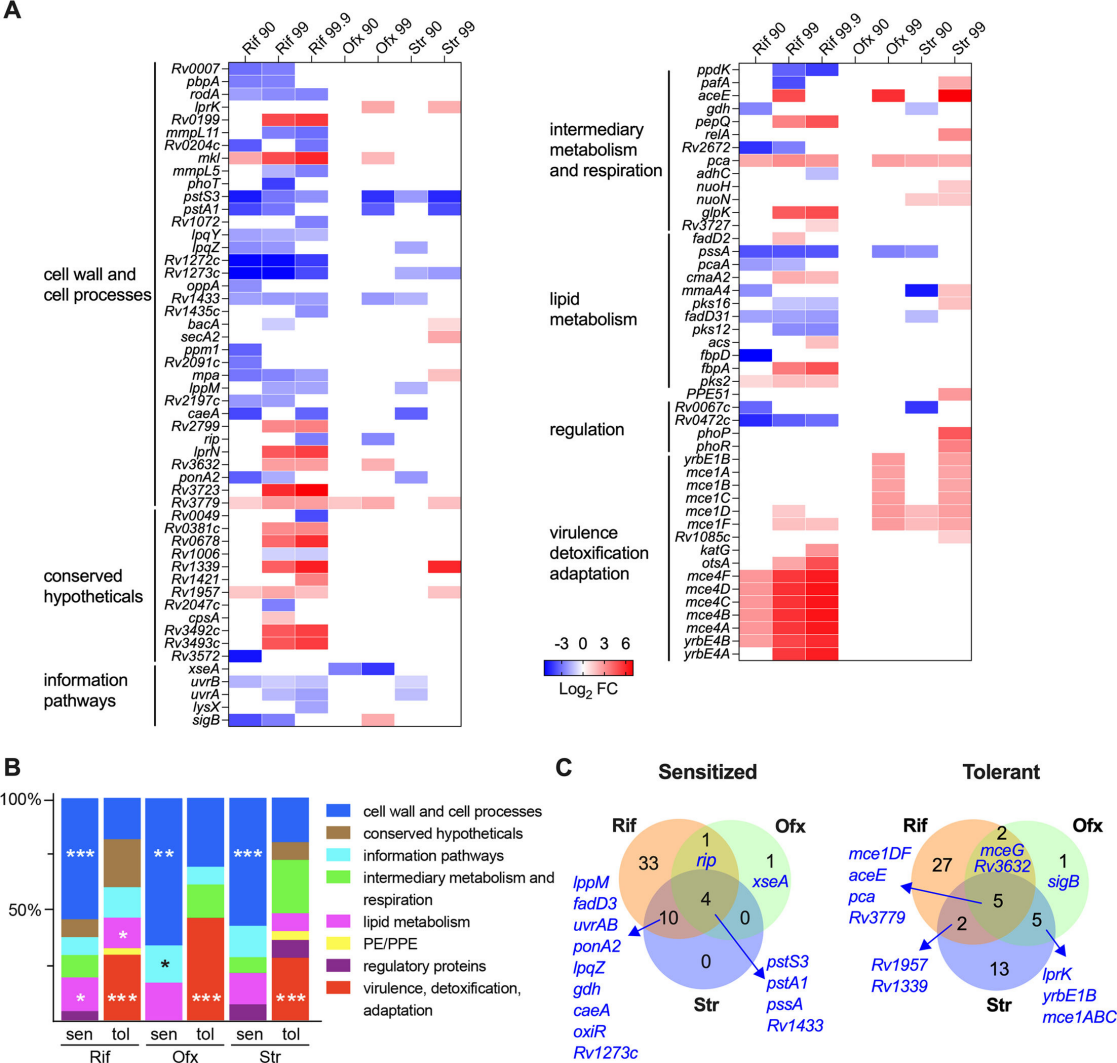
Mutants with altered antibiotic efficacy. (**A**) Heatmap showing the log_2_FC of the identified mutants with altered antibiotic efficacy. Data with non-significant change were shown as white cells. (**B**) Functional categories of the identified genes. (**C**) Venn diagrams showing the number of genes associated with altered sensitivity to Rif, Str, and Ofx.

Analyses of the Tn-seq data derived from seven drug regimens identified 98 mutants that exhibited different abundance in input (S0) and posttreatment pools (Fig. 3A and Table S4). Many genes known to influence antibiotic efficacy were identified in this study. For example, multiple mutants lacking antibiotic efflux pumps, including ABC transporters Rv1273-Rv1274 (34), MmpL5 (35), PstA1-PstS3 (36), and BacA (34), were more susceptible to Rif and/or Str. Conversely, we found that the *glpK* (16, 17)*, rv1339* (15)*, pepQ* (35), *ppe 51* (37), *phoPR (38*), *mceG* (rv0655, known also as *mkl*) (38, 39), and *cmaA2* (40) mutants became less sensitive to Rif or Str, consistent with prior studies showing that mutations in these genes were associated with drug resistance or tolerance. These results indicate that the Tn-seq-based screen can provide an accurate assessment of relative mutant abundance during antibiotic treatment.

Functional categories related to cell wall and cell process were significantly enriched in the mutants showing increased sensitivity (decreased Log_2_FC) to Rif, Str, and Ofx (Fig. 3B and Table S4), suggesting that drug entry across cell permeability barriers is a primary determinant of antibiotic efficacy. Among the mutants tolerant to killing (increased Log_2_FC) by all three antibiotics, genes belonging to the virulence, detoxification, and adaptation categories were significantly enriched (Fig. 3B and Table S4). These genes mainly encode components of the Mce1 and Mce4 complexes, which have been characterized as importers involved in transport of host-derived lipids during infection (39, 41). Moreover, genes related to lipid metabolism were specifically enriched in the mutants with altered sensitivity to Rif (Fig. 3B).

We next analyzed drug-specific effects of these drug efficacy-altering genes (Fig. 3C and Table S4). Most insertion mutants (84 genes) had significantly changed fitness to Rif, while only 39 and 19 mutants altered Str and Ofx sensitivity. We identified 60 mutants that only altered Rif susceptibility. The largest overlap, 21 genes, was observed between Rif and Str (Fig. 3C). Nine mutants exhibited changed sensitivity to all three drugs, including four sensitized mutants that contain insertion mutations in *pstS3*, *pstA1*, *pssA* (encoding a phosphatidylserine synthase), and *rv1433*, and five drug tolerance-conferring mutants of *mce1D, mce1F*, *aceE*, *pca*, and *rv3779* (Fig. 3C). Intriguingly, these sensitized mutants were all defective in genes encoding the cell membrane and cell wall components. Previous studies showed that expression of the *pst*S3C2A1 operon can be induced by phosphate starvation, and the mutants deficient in this system were sensitive to multiple stresses including phosphate depletion, hypoxia, sodium dodecyl sulfate, and reactive oxygen and nitrogen species (42). Rv1433 was predicted to be homologous to L, D-transpeptidase 3 (PFAM# O06825) that may perform unknown peptidoglycan cross-linking reactions. Finally, our results also revealed cellular activities that had opposite effects on drug efficacy (Table S4). For example, mutants with insertions in *pafA* and *mpa*, both of which encode the *Mtb* proteasome factors, exhibited contrasting effects in Rif and Str sensitivity (Fig. 3A and Table S4).

### The correlation between stationary phase adaptation and antibiotic susceptibility

**Fig 4:**
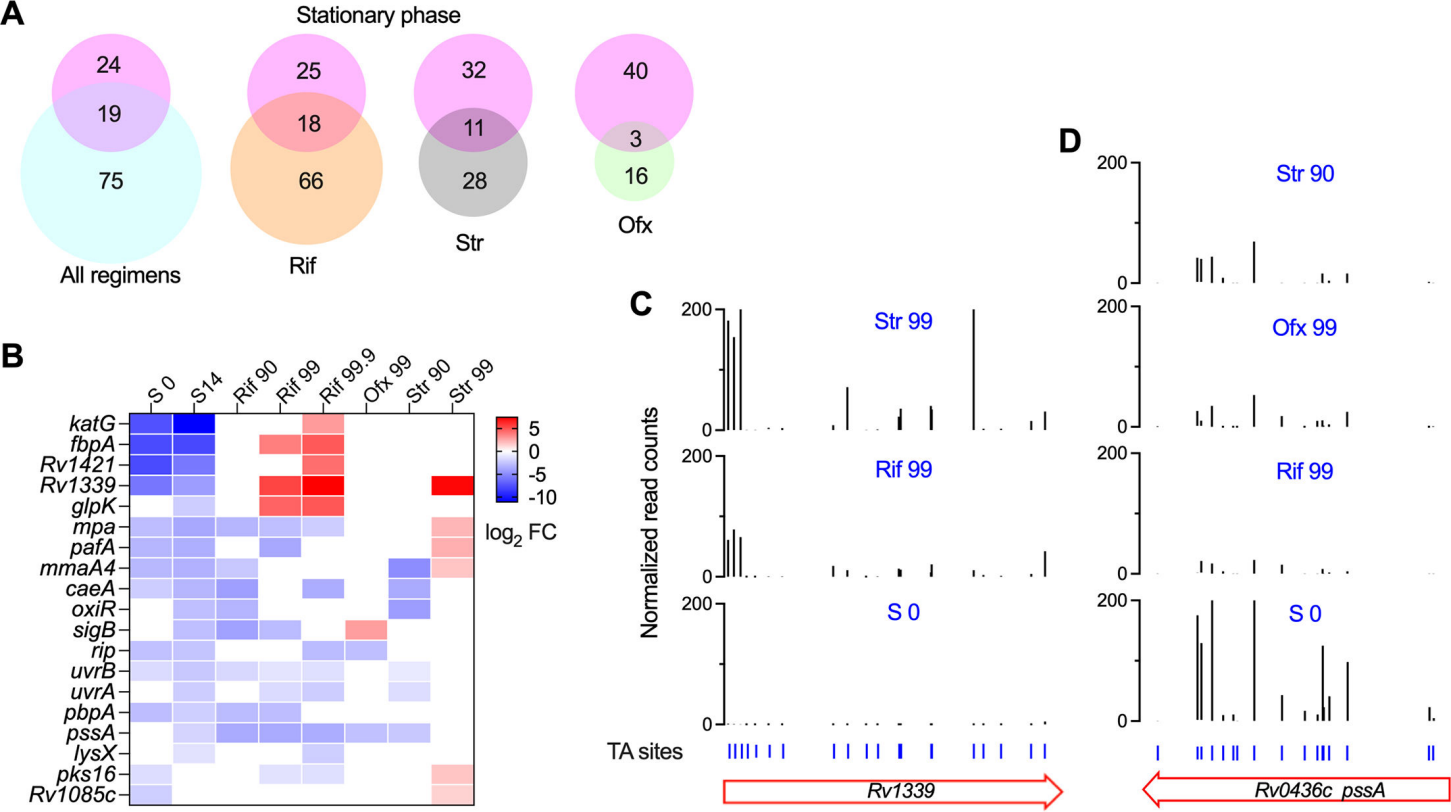
Correlation between stationary phase adaptation and antibiotic susceptibility. (**A**) Venn diagrams showing the number of genes associated with stationary phase survival and antibiotic efficacy. (**B**) Heatmap showing the log_2_FC of the identified mutants during the stationary phase and drug exposure. Data with non-significant change were shown as white cells. (**C–D**) Comparison of transposon insertion abundances at different conditions (S0, Rif 99, Str 90, and Ofx99).

To further investigate the mechanisms between stationary phase adaptation and antibiotic susceptibility, we compared the fitness of each mutant during the stationary phase and antibiotic exposure. Among the 43 mutants exhibiting reduced fitness during the stationary phase, 19 mutants had altered drug susceptibility, demonstrating that most of the mutants that failed to maintain a nongrowing state did not directly alter drug efficacy (Fig. 4A). Notably, nearly all these 19 mutants exhibited altered fitness during Rif treatment (Fig. 4A and B), indicating that Rif extensively interferes with cellular activities underlying stationary phase adaptation, which is consistent with the notion that Rif action is a primary determinant of the sterilization effect of the standard TB regimen against nongrowing Mtb (2, 3). We observed a small overlap of genes associated with both stationary phase adaptation and altered sensitivity to Str (11 genes) or Ofx (three genes).

We found that many mutants that compromise stationary phase adaptation resulted in increased fitness during antibiotic treatment (Fig. 4A and B). For example, while mutants with insertions in *katG*, *fbpA*, *rv1421*, and *rv1339* showed markedly reduced fitness (log_2_FC < -4.3) during the stationary phase, they were profoundly overrepresented (log_2_FC > 2.9) during Rif treatment (Fig. 4C). A recent study demonstrated that variations in Rv1339 confer slow growth on glycerol and propionate and multidrug tolerance in clinical Mtb strains (15), which are highly consistent with the Tn-seq phenotypes of the *rv1339* mutants. Among these genes, *katG* is a well-established INH resistance gene, and mutations that reduce KatG catalase-peroxidase activity can lead to high levels of INH resistance (35); e.g., the most prevalent KatG S315T mutation caused 50% reduction in the catalase-peroxidase activity and resulted in a 200-fold increase in INH MIC (43). Interestingly, a prior study found that KatG S315T overwhelmingly arose before mutations that confer Rif resistance across all Mtb lineages, geographical regions, and time periods (44). Our Tn-seq results suggest that mutations that disrupt KatG function may decrease Rif efficacy against nongrowing Mtb.

Our result showed that inactivation of *caeA*, *oxiR*, *rip*, *uvrAB*, *pbpA*, *pssA*, and *lysX* concurrently impaired the survival ability of Mtb during the stationary phase and sensitized the bacilli to antibiotics (Fig. 4B). Notably, mutants with insertion mutations in *caeA*, *rip*, *uvrAB*, and *pssA* also exhibited reduced survival in hypoxia conditions (Fig. 2F), suggesting potential targets for drug development. Among these genes, the *pssA* mutant was the only one that could sensitize nongrowing Mtb to all tested drugs (Fig. 4D). Studies in *Escherichia coli* found that PssA functions as a phosphatidylserine synthase responsible for phosphatidylethanolamine (PE) biosynthesis (45). Overexpression of *pssA* in *E. coli* increased the abundance of membrane PE and significantly changed membrane integrity, electrochemical potential, and hydrophobicity, resulting in markedly increased resistance to membrane-damaging solvents (46). These results collectively suggest a crucial role of the modification of membrane phospholipids in bacterial adaptation to growth-limited conditions.

### Mutations in Mce1 and Mce4 cause drug tolerance

**Fig 5:**
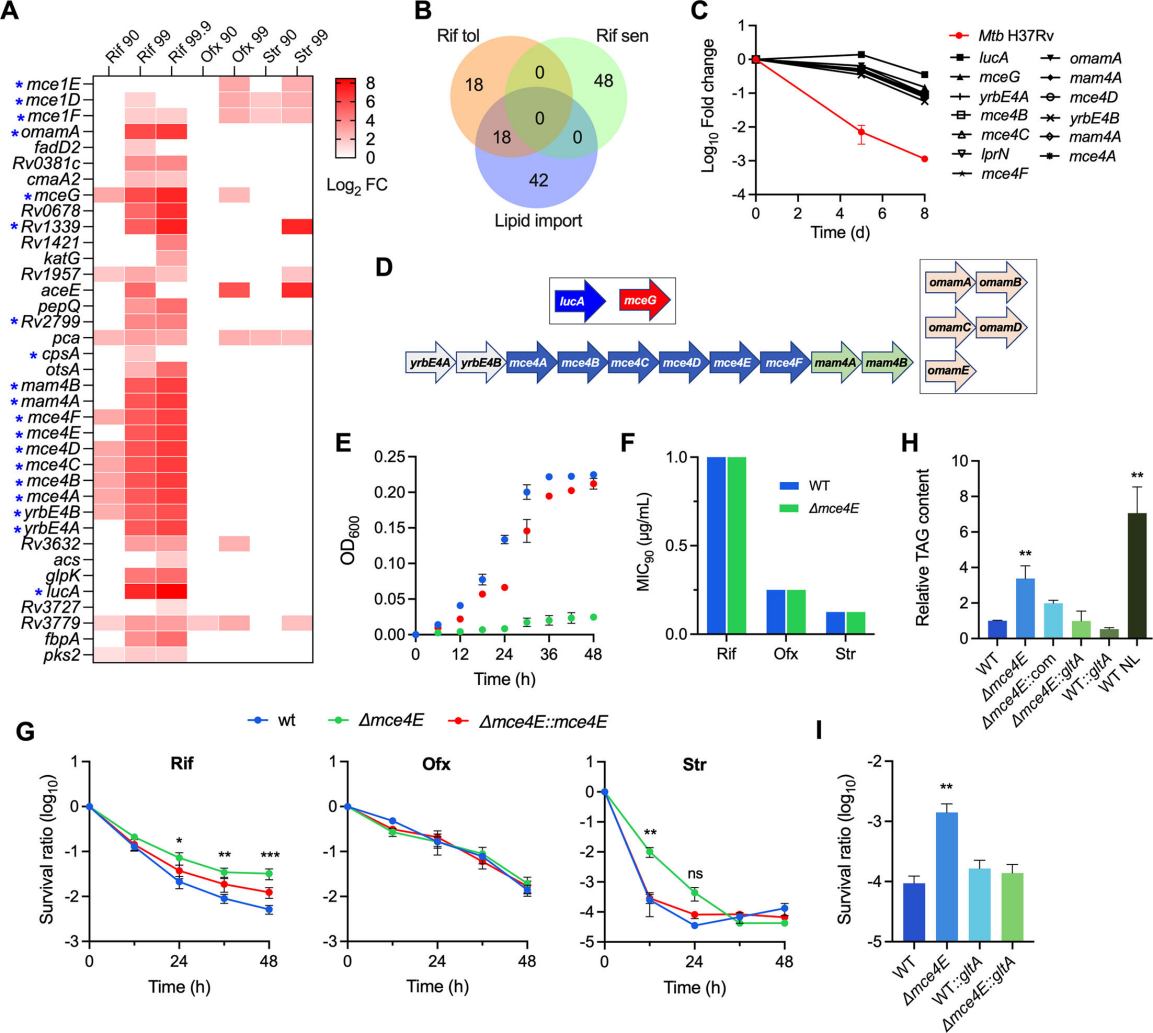
Mutations in Mce1 and Mce4 cause drug tolerance. (**A**) Heatmap showing the log_2_FC of the identified mutants refractory to Rif killing. Genes associated with lipid import are marked with an asterisk. Data with non-significant change were shown as white cells. (**B**) Venn diagrams showing the number of genes associated with Rif sensitivity and lipid import. (**C**) Tn-seq phenotypes of the *mce4* mutants significantly overrepresented following exposure to Rif. (**D**) Components and operon organization of the Mce4 system. (**E**) Growth curves of the indicated strains in minimal media supplemented with 266 μg/mL cholesterol. (**F**) MIC_90_ measured with 7H9 supplemented with glycerol. (**G**) Time-kill curve of indicated strains treated with 50 µg/mL Rif, 5 µg/mL Ofx, and 1.25 µg/mL Str. Strains were cultured in 7H9 supplemented with glycerol. (**H**) TAG content measured from the indicated strains cultured 7H9. NL, a positive control treated by Nitrogen Limiting. (**I**) Survival of the indicated strains after treated with 50 µg/mL Rif for 72 h. Data shown are mean ± SEM from at least three independent experiments (**E–G**). *P* values were determined using an unpaired *t*-test. *P < 0.05, **P < 0.01, and ***P < 0.001.

Increasing evidence is showing that the host microenvironments, particularly drug exposure, can select Mtb mutations that are refractory to antibiotic treatment (15, 38, 47). We next focused on the mutants that cause increased fitness during Rif treatment (a sterilization agent against nongrowing Mtb) (Fig. 5A). Strikingly, we found that over half of these genes are associated with lipid metabolism, including lipid import (Mce1 and Mce4 complexes) and catabolism (FadD2, Acs, GlpK, and Rv1339) (Fig. 5A). According to the findings of a prior genome-wide screen study (39), half of these Rif-refractory mutants were deficient in lipid import (Fig. 5A and B). Remarkably, nearly all the genes encoding the Mce4 importer were identified in our study, and mutations in these genes markedly increased bacterial fitness (log_2_FC > 6) during Rif treatment (Fig. 5C and D).

Mce4 is an importer for the utilization of host-derived cholesterol during Mtb infection and was found to participate in the response to the stress conditions by controlling lipid homeostasis of the cell wall (41, 48). To validate the Tn-seq results, we constructed a *mce4E*-null mutant of *Mycobacterium smegmatis*, in which the Mce4 has a transporter function similar to that of Mtb (48, 49). Consistent with prior observations (48–50), the ∆*mce4E* mutant exhibited a growth defect when cholesterol was supplemented as the sole carbon source, suggesting a deficiency in importing cholesterol (Fig. 5E). Our results showed that the inactivation of Mce4 had no influence on the MIC of Rif, Str, and Ofx (Fig. 5F). In agreement with the Tn-seq phenotype, inactivation of Mce4 increased bacterial survival during Rif treatment but did not affect Ofx lethality (Fig. 5G). Upon exposure to Str, the ∆*mce4E* mutant exhibited a significant delay in the rate of killing but did not influence overall cell death relative to wild-type *M. smegmatis* (Fig. 5G). Altogether, these results demonstrate that mycobacterial cells deficient in Mce4 are tolerant to Rif killing.

Interestingly, the Rif-sensitive phenotypes of both the *mce4E*-null *M. smegmatis* and the *mce4*-deficient Mtb occurred in culture conditions without cholesterol (Fig. 5C and G), suggesting cholesterol metabolism may not contribute to this phenotype. Consistent with prior observations in *M. smegmatis* and *Mycobacterium avium paratuberculosis* (51, 52), we found that deletion of *mce4E* resulted in triacylglycerol (TAG) accumulation (Fig. 5H). To investigate whether TAG accumulation affects Rif susceptibility, we overexpressed citrate synthase *gltA*, which could effectively compete for acetyl-CoA and thus decrease the generation of TAG in *Mtb* (12). The results showed that *gltA* overexpression markedly reduced TAG content in the *mce4E*-null *M. smegmatis* and restored the survival defect upon exposure to Rif (Fig. 5H and I), suggesting that TAG accumulation caused by Mce4 inactivation is a causative reason of Rif tolerance.

### The activity of lipid metabolism influences Rif susceptibility

**Fig 6:**
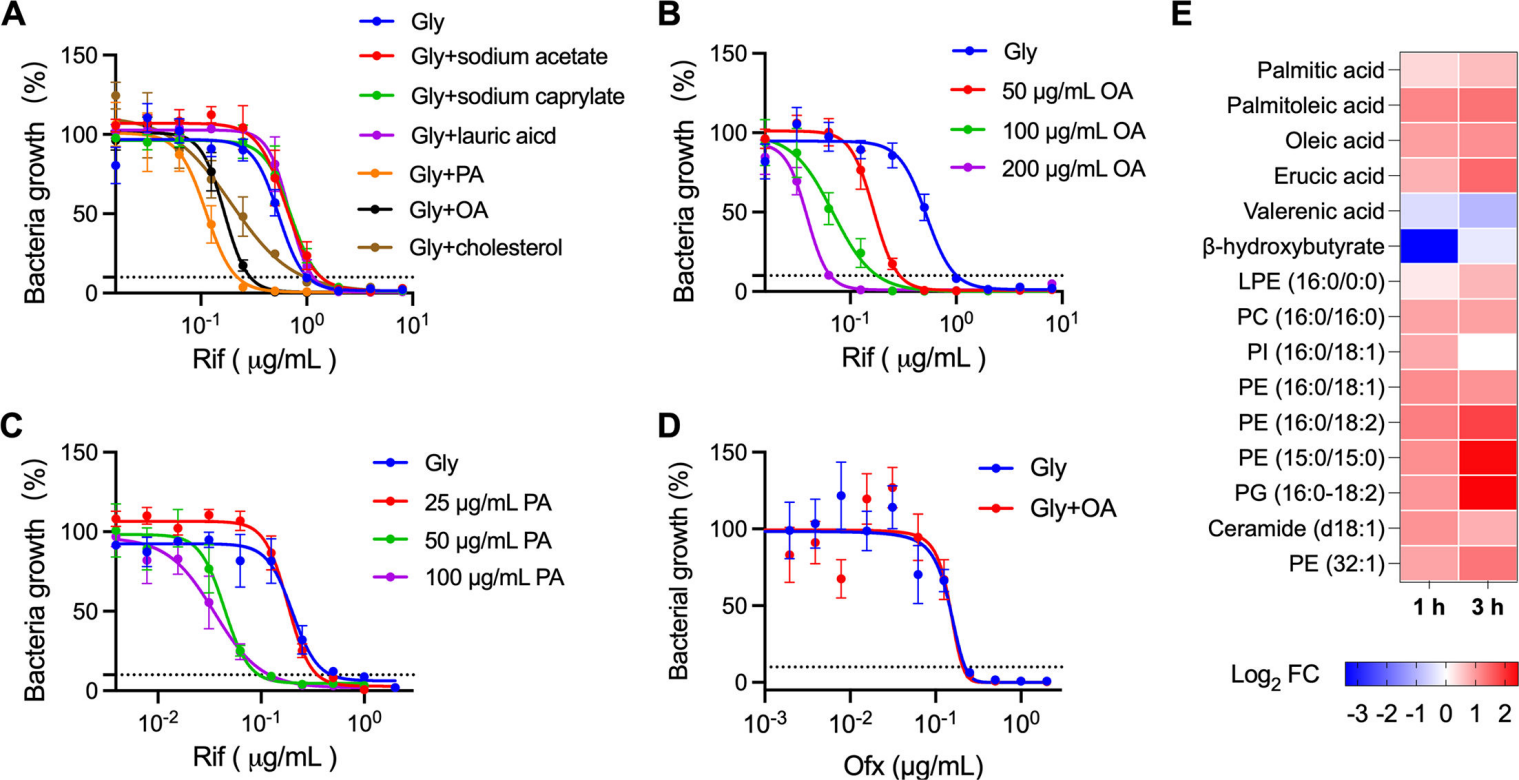
Lipid metabolism is associated with Rif susceptibility. (**A–D**) Antibiotic MIC against *M. smegmatis* measured in the 7H9 media supplemented with the indicated carbon sources. Gly, glycerol; OA, oleic acid; PA, palmitic acid. (**E**) Heatmap of the identified lipid metabolites with a significance in prediction (VIP) score >1 and a *P*-value < 0.1. *M. smegmatis* cells collected at indicated time points post-treatment with 16 µg/mL Rif were subjected to LC-MS/MS-based metabolomics. Data shown are Log_2_FC normalized to T = 0. Data with non-significant changes were shown as white cells. PE, phosphatidylethanolamine; PI, phosphatidylinositol; PG, phosphatidylglycerol; PC, phosphatidylcholine.

Given the profound enrichment of genes involved in lipid metabolism in the identified mutants that are refractory to Rif killing, we sought to assess whether lipid metabolism influences Rif susceptibility. To this end, we first measured the drug susceptibility under growth conditions with or without the supplementation of fatty acids (Fig. 6A). We found that the presence of long-chain fatty acids, such as palmitic acid (C16) or oleic acid (OA, C18), in the 7H9 media (containing 0.2% glycerol) reduced the Rif MIC against *M. smegmatis* in a concentration-dependent manner (Fig. 6A through C). This effect was not observed with cholesterol and short-chain fatty acids such as acetate (C2), caprylate (C8), and lauric acid (C12) (Fig. 6A). Interestingly, we found that the supplementation with OA did not alter Ofx MIC (Fig. 6D), suggesting that long-chain fatty acid-induced sensitization is Rif-specific and may not result from increased cell wall permeability. These results indicate that the availability of environmental long-chain fatty acids influences Rif susceptibility.

To gain further insights into whether Rif action is associated with mycobacterial lipid metabolism, we used liquid chromatography-tandem mass spectrometry (LC-MS/MS)-based metabolomics and identified 15 lipid metabolites showing changed cellular concentrations following Rif treatment (Fig. 6E). Our results showed that Rif treatment significantly increased the cellular concentrations of long-chain fatty acids, including erucic acid, palmitic acid, palmitoleic acid, and OA, but reduced the levels of short-chain fatty acids such as valerenic acid and β-hydroxybutyrate (Fig. 6D). Moreover, Rif treatment also significantly changed cell membrane phospholipid composition, particularly the positively changed phosphatidylethanolamine and phosphatidylcholine (Fig. 6E). Altogether, these results provide evidence that lipid metabolism is associated with Rif action.

## DISCUSSION

The requirement of prolonged treatment for tuberculosis patients is attributable to the presence of slowly growing or nongrowing Mtb (3, 4). Although the genetic determinants of Mtb adaptation to growth-limited conditions, such as hypoxia (12, 33) and acid stress (27), have been extensively identified, it remains unclear to what extent the cellular processes necessary to sustain the Mtb nongrowing state affect drug efficacy. In this study, we performed a genome-wide transposon-mediated screen, which allowed parallel identification of the genes that influence bacterial fitness and drug efficacy during the stationary phase. Our analyses provide the detailed assessment of Mtb genes necessary for adaptation to the stationary phase and drug treatment.

Our screen yielded 43 mutants showing reduced fitness during the stationary phase. Genes related to SOS response, proteolysis, phospholipid biosynthesis, response to antibiotic and nitrosative stress, and acid resistance were significantly enriched among the identified mutants. Among the 43 genes, 25 genes were found to contribute to Mtb adaptation to hypoxia (33), suggesting that these genes are generally required for Mtb adaptation to growth-limited conditions. An interesting observation is that many mutants exhibited opposite fitness changes in the stationary phase and hypoxia conditions, including the recently identified genetic markers *glpK* and *Rv1339* associated with poor clinical outcomes (15–17). These results suggest that the nongrowing Mtb cells induced by different growth-limiting conditions may have distinct adaptation strategies. In this regard, it is noteworthy that the nongrowing Mtb cells during infection are formed in response to multiple host-derived stresses (53), and increasing evidence is showing that the environmental heterogeneity *in vivo* has great impacts on the treatment outcomes in real infections (54). Thus, the ultimate role of the identified genes in pathogenicity and antibiotic efficacy still requires further investigation.

Tuning the capacity of gene expression to match limited substrates is an inevitable process during the stationary phase (23). Alternative sigma factors are transcriptional regulators responsible for orchestrating the gene expression responses to growth-limiting conditions. Prior studies found that the sigma factor genes *sigF* and *sigB* are induced during the stationary phase (55, 56). Our results showed that the *sigB* mutants exhibited reduced fitness (log_2_FC = -2.8) during the stationary phase, suggesting a crucial role of SigB in Mtb’s transition to the stationary phase. We also found that the transcriptional regulators FdmR (Rv0238) (57) and OxiR (Rv0067c) (58) are required for Mtb adaptation to the stationary phase. FdmR was characterized as a long-chain acyl-coenzyme A-responsive repressor of genes involved in fatty acid degradation and modification (57). Inactivation of FdmR resulted in overactive catabolism of fatty acids and synthesis of shortened lipids including cell wall component phenolic glycolipid (PGL), TMM, and TDM (57). These results suggest that regulation of lipid homeostasis plays a critical role in adaptation of Mtb to the stationary phase. Finally, the reduced fitness of mutants deficient in GreA, which can help to rescue backtracked RNA polymerases (59), indicates that RNA polymerases pausing and arrest in severely substrate-limited conditions is detrimental to the adaptation of Mtb to the stationary phase.

We identified 98 mutants that altered antibiotic efficacy in stationary phase Mtb. Many genes known to influence antibiotic efficacy were identified in this study, demonstrating that the Tn-seq-based screen can provide an accurate assessment of relative mutant abundance during antibiotic treatment. Our analyses indicated that the reduced drug entry is a critical factor that limits antibiotic efficacy in nongrowing Mtb. For example, genes involved in cell envelope biosynthesis and remodeling (*caeA*, *rip*, *pbpA*, *pssA*, *lysX*, *lppM*, *ppm1*, *pcaA*, *lpqYZ, mmpL11, rodA, rv1433,* and *rv1421*), antibiotic efflux (*rv1273-rv1274*, *mmpL5*, and *bacA*), and phosphate transport (*pstA1-pstS3*) are significantly enriched in the mutants sensitized to antibiotic killing. Our results also revealed cellular activities that had opposite effects on drug susceptibility. For example, mutants with insertions in *pafA*, *mpa,* and *mmaA4* exhibited contrasting effects in Rif and Str sensitivity. These results suggested that different antibiotics are limited by distinct factors. Thus, it will be interesting for future studies to uncover the cellular pathways that influence the susceptibility of nongrowing Mtb to other drugs such as the newly approved bedaquiline and delamanid.

We found that genes involved in lipid metabolism (39, 60), including lipid import (Mce1 and Mce4 complexes) and catabolism (FadD2, Acs, GlpK, and Rv1339), were specifically enriched in the mutants with decreased sensitivity to Rif, demonstrating a drug-specific effect of Mtb metabolism. We validated that the mutant deficient in *mce4E* exhibited increased survival during Rif treatment without changing its MIC, indicating that the Mce4 mutation causes drug tolerance. However, given that the Mce4 system plays an essential role in importing cholesterol and Mtb pathogenicity in mice (40, 41), mutations in this system may not be selected during infection due to severe fitness cost. Furthermore, we found that the presence of palmitic acid or OA in the 7H9 media (containing 0.2% glycerol) reduced the Rif MIC in a concentration-dependent manner, suggesting the availability of environmental long-chain fatty acids influences Rif susceptibility. In accordance with these observations, Rif treatment significantly perturbs lipid metabolism of mycobacterial cells. Overall, these results indicate that the activity of lipid metabolism has a great impact on Rif susceptibility.

We found that only a small fraction (19/43) of mutants that compromise stationary phase adaptation of Mtb had altered drug susceptibility, and nearly all these mutants exhibited altered fitness during Rif treatment, suggesting that failure to adapt to a nongrowing state does not necessarily alter drug sensitivity. Moreover, our results showed that mutations in *katG*, *fbpA*, *rv1421*, *glpK*, and *rv1339*, which markedly reduced bacterial fitness during the stationary phase, were profoundly overrepresented (log_2_FC > 2.9) during Rif treatment. These results are consistent with those of recent studies showing that mutations in *glpK* and *rv1339* contribute to drug insensitivity of clinical Mtb isolates (15–17). In line with the published Tn-seq screen and *katG* deletion results (26, 31), we found that transposon inactivation of *katG* did not affect bacterial fitness during exponential growth (Table S2). Thus, the markedly reduced fitness of the *katG* mutants during the stationary phase suggests that detoxification of endogenous peroxides is essential for Mtb adaptation to growth-limited conditions. Recent studies found that the INH-resistant KatG mutants sensitize multidrug-resistant Mtb to the newly approved anti-tuberculosis drugs (bedaquiline, pretomanid, Q203, and linezolid) (61–63) but did not affect the MIC and killing efficacy of Rif against exponential-phase Mtb (61). In contrast to these observations, our results showed that KatG deficiency decreases Rif efficacy against nongrowing Mtb, suggesting that the KatG deficiency-induced collateral drug sensitivity may rely on the states of bacterial growth and metabolism.

We identified eight mutants that concurrently impaired the adaptation of Mtb to the stationary phase and sensitized the bacilli to antibiotics, suggesting potential targets for drug development. Notably, genes involved in cell membrane biosynthesis and remodeling (*caeA*, *rip*, *pbpA*, *pssA*, and *lysX*) were significantly enriched among these mutants. Further studies are needed to elucidate the mechanisms of cell envelope biosynthesis and remodeling in nongrowing Mtb.

## MATERIALS AND METHODS

### Strains and growth conditions

Mtb H37Rv (The American Type Culture Collection, 27294) and *M. smegmatis* mc^2^155 (The American Type Culture Collection, 706) were used in this study. Mycobacterial strains were grown in Difco Middlebrook 7H9 broth (BD #271310) or on 7H10 (BD #262710) agar supplemented with 0.5% glycerol, 0.05% Tween 80, and 10% OADC (*Mtb*). Where appropriate, hygromycin B and kanamycin were added at 50 and 20 µg/mL, respectively. Experimental cultures were started by inoculating the overnight culture into fresh medium (without antibiotic) to achieve an OD_600_ of 0.01 ~ 0.02 and then incubated at 37°C with shaking at 100 rpm. The beginning of the stationary phase of growth was defined as a cease in increasing OD_600_ for 1 d (S0).

### Generation of the transposon mutant library

A himar1-transposon mutant library was generated in Mtb by transduction with phiMycoMarT7(24). Transformants were grown on 7H10 agar supplemented with kanamycin (50 μg/mL) and comprised approximately 200,000 independent insertion events. The kanamycin-resistant colonies were pooled in 7H9 containing 12% glycerol and stored at −80°C.

### Library screening

Approximately 10^6^ colony-forming units (CFUs) of library were inoculated into 100 mL 7H9 in a 500 mL flask at 37°C. Aliquots of 2 million CFUs were collected at S0 and S14, plated onto ten 7H10 plates (20 mm diameter, corresponding to 0.2 million CFUs per plate), supplemented with kanamycin, and incubated at 37°C for 28 d. Colonies were pooled in 10 mM Tris-HCl 1 mM EDTA and stored at −80°C. The S0 culture was aliquoted and exposed to 12.5 μg/mL Rif, 30 μg/mL Ofx, or 10μ g/mL Str. At indicated time points (Fig. 1D), approximately 2 million CFUs were collected by centrifugation at 4,000 × *g* for 10 min, washed with fresh 7H9 broth to remove residual antibiotic, and then recovered by plating, as described above. Two biological replicates were performed.

### Tn-Seq

Harvested cells were prepared for Tn-Seq, as previously described (24). Briefly, genomic DNA was extracted from cell pellets using the TIANamp bacteria DNA kit (Tiangen). DNA was quantified and mechanically sheared by ultrasonication. Sheared DNA fragments were subjected to end repair (Epicenter) and dA-tailing with Taq polymerase to allow ligation of barcoded adapters 5′-ATGATGGCCGGTGGATTTGTGNNANNANNNTGGTCGTGGTAT-3′ and 3′-NH_2_-ACCAGCACCAT-5′. The Tn-chromosome junction regions were amplified for 20 cycles (95°C, 30 sec; 58°C, 20 sec; 72°C, 30 sec) using adapter primer 5′-ATGATGGCCGGTGGATTTGTG-3′ and himar1 primer 5′-TAATACGACTCACTATAGGGTCTAGAG-3′. After PCR purification, Illumina-specific adapter sequences were added through an additional PCR (20 cycles of 95°C, 30 sec; 58°C, 20 sec; 72°C, 45 sec) before sequencing using 5′-AATGATACGGCGACCACCGAGATCTACACTCTTTCCCTACACGACGCTCTTCCGATCTCGGGGACTTATCAGCCAACC-3′ and 5′-CAAGCAGAAGACGGCATACGAGATTGTTTCGAGTGACTGGAGTTCAGACGTGTGCTCTTCCGATCTGTCAATGATGGCCGGTGGATTTGTG-3′.

### Sequence analysis

Raw sequencing reads were first trimmed to remove the transposon sequence; reads with a flanking genome region longer than 20 bp were retained. Then, trimmed reads with proper indexes were mapped to a unique site on the Mtb H37Rv genome (NCBI, NC_000962). Finally, mapped reads were filtered by their indexes to eliminate PCR bias. Those insertion sites in the genome and the count of each read were recorded. Pearson’s correlation and distribution of reads were calculated and visualized with Python and R. Fitness change was determined by carrying out a pairwise comparison of each sample with the input data. Reads in the 5% N-terminal and 5% C-terminal of the gene sequence were discarded. Read counts, P-value determined by the permutation test (adjusted by using the method of FDR), and log_2_FC between the input and post-treatment were calculated. The genes with an adjusted *P*-value of less than 0.05 and log_2_FC of more than 1 or less than −1 were selected.

### Construction of knockout and complementation strains

The *M. smegmatis Δmce4E* strains were constructed through allelic exchange using the pYUB854 plasmid (64). To introduce the *mce4e* deletion in *M. smegmatis*, homologous arms flanking the *mce4E* gene were first PCR-amplified using left arm primers (5′-CTTAAGGCCTTGACTAGAGGGTACGAGGCCGAAGTCGCCATGAC-3′ and 5′-AGATACCTAGGTGAGCTCTGGTACCCCCAGGCGGGTTCCCTTAC-3′) and right arm primers (5′-CTAGCA CGCGCACCATGGGAAGCTATGATCGACCGGCTGACACG-3′ and 5′-CAGGATATCTGGATCCA CGAAGCTCGAAGACCCCACTGTGCTGA-3′), and the amplicons were gel-extracted and cloned into the pYUB854 plasmid using the recombination method (Beyotime, D7010M). The resulting plasmid was electroporated into wild-type *M. smegmatis* cells. Hygromycin B-resistant mutant clones were selected and confirmed by PCR and Sanger sequencing. For complementation of the *M. smegmatis* gene-deletion strains, wild-type *mce4E* was PCR-amplified using 5′-CGCAATGGCTAAGACAATTGCCATGCGCACCCTGGCGATCGGCAG-3′ and 5′-TTAACTACGTCGACATCGATAAGCTCTACGGCCCCTCGTGCTGC −3’) and cloned into an integrative plasmid pMV361 containing a constitutive groL1 promoter (47). The citrate synthase *gltA* (*MSMEG_5672*) was PCR-amplified using 5′- CAATTGCGGATCCAGCTGCAGAATTGATGAAGGGATTCCCGTGGCCG-3′ and 5′- TTAACTAC GTCGACATCGATAAGCTGGTGAAACCGGTTCAGCGG −3’) and cloned into pMV261.

### Cholesterol growth assay

Cholesterol stock solution was prepared by dissolving cholesterol in cyclodextrin (65). The cholesterol growth assay was adapted from previous studies (50). In brief, *M. smegmatis* strains were washed twice by M9 medium by centrifugation at 2,000  × *g* for 1  min at 22°C. After the wash steps, strains were resuspended in 15 mL M9 medium (in 100 mL flasks) with 0.04% tyloxapol to an OD_600_ of 0.01. For each strain, the following media were used: (1) M9  + 0.04% tyloxapol +9  mg/mL methyl-β-cyclodextrin (MBC) (no carbon source control); and (2) M9  + 0.04% tyloxapol + 9 mg/mL MBC  + 0.69  mM cholesterol. The cultures were grown at 37°C 120 rpm, and the OD_600_ was monitored every 6 h.

### Antibiotic killing assay

Strains were grown in 7H9 broth or on 7H10 agar. Three colonies were inoculated into 3 mL medium and grown at 37°C, 120 rpm to reach a cell density of OD_600_ ~2 and then diluted it in 10 mL 7H9 media in 50 mL flasks with an initial OD_600_ of 0.01. At an OD_600_ of 0.4 to 0.5, aliquots of 1 mL culture (in a 14 mL tube) were treated with antibiotics at 37°C. Samples were diluted and then plated onto 7H10 agar plates to determine the CFU. Survival was expressed as the ratio compared with pretreatment. At least three biological replicates were conducted.

### Antibiotic susceptibility

To measure the MIC, strains were grown to the exponential phase and diluted to ~10^5^ CFU per mL in 200 µL 7H9 media containing antibiotics at indicated concentrations. 7H9 media with 0.2% glycerol and supplemented with or without 0.005% sodium acetate, 0.005% sodium caprylate, 0.00125% lauric acid, 0.005% palmitic acid, 0.005% oleic acid, or 0.01% cholesterol as an additional carbon source. Cells were cultured in a 96-well plate at 37°C to allow the untreated control to grow to an OD_600_ of 1.0. The bacterial growth was monitored by OD_600_ and expressed as a percentage of growth relative to the untreated control. The growth percentage-antibiotic concentration curve was drawn to determine the 90% inhibitory concentration (MIC_90_).

### Measure of TAG

Cellular TAG contents were measured by Nile Red staining and fluorescence microscopy, as previously described (66). Strains were cultured at 37°C, 120 rpm until the OD_600_ reached ~0.4. To set up the positive control, the wild-type *M. smegmatis* culture was washed with PBS and then transferred to Mineral Salt Medium Nitrogen Limiting (66) (2 g/L Na_2_HPO_4_, 1 g/L KH_2_PO_4_, 0.5 g/L NaCl, 0.2 g/L MgSO_4_, 20 mg/L CaCl_2_, 0.05 g/L NH_4_Cl, 1% glycerol, and 0.02% (vol/vol) Tyloxapol). Cells from 2 mL culture were centrifuged at 12000 × *g* for 30 s, washed twice with PBS, and resuspended in 300 µL of PBS. The cell suspension was mixed with 15 µL Nile red (0.5 mg/mL, ethanol dissolved) and incubated at 37°C in the dark for 20 min. Cells were collected by spinning at 12,000 × *g* for 30 s, washed twice with PBS, and resuspended in 200 µL of PBS. Cells were dropped onto a slide, and the snapshot imaging analysis of bacteria was performed using fluorescence confocal microscopy (Nikon C2) at room temperature.

### Metabolomics

*M. smegmatis* was cultured to an OD_600_ of 0.3–0.4 and then treated with 16 µg/mL Rif. Cells from 50 mL culture were collected by centrifugation at 12,000 × *g* for 1 min at diﬀerent time points after Rif treatment. Cells were washed once with pre-warmed PBS (37°C) before adding 800 µL of cold methanol/acetonitrile (1:1, vol/vol). The mixture was centrifuged at 14,000 × *g* for 5 min. The supernatant was dried in a vacuum centrifuge. For LC-MS analysis, the samples were re-dissolved in 100 µL acetonitrile/water (1:1, vol/vol) solvent. Samples were analyzed using a 2.1 mm × 100 mm ACQUIY UPLC BEH 1.7 µm column (Waters, Ireland). In both ESI-positive and -negative modes, the mobile phase contained solvent A, which was 25 mM ammonium acetate and 25 mM ammonium hydroxide in water and solvent B, which was acetonitrile. The gradient was 85% B for 1 min and was linearly reduced to 65% in 11 min, then was reduced to 40% in 0.1 min and kept for 4 min, and then increased to 85% in 0.1 min, with a 5 min re-equilibration period employed. The ESI source conditions were set as follows: Ion Source Gas1 as 60, Ion Source Gas2 as 60, curtain gas as 30, source temperature: 600°C, and IonSpray Voltage Floating (ISVF) ±5,500 V. In MS-only acquisition, the instrument was set to acquire over the *m/z* range 60–1000 Da, and the accumulation time for TOF MS scan was set at 0.20 s/spectra. In auto MS/MS acquisition, the instrument was set to acquire over the *m/z* range 25–1,000 Da, and the accumulation time for product ion scan was set at 0.05 s/spectra. The detected metabolites were characterized according to Metabolomics Standards Initiative standards. The metabolites were confirmed to be at level 2 or above by matching the retention time, molecular mass (within <25 ppm), secondary fragmentation spectra, and collision energy of the metabolites in the local database. Metabolites with a significance in prediction (VIP) score greater than 1 and a P-value less than 0.1 for the OPLS-DA variable were considered significantly diﬀerent.

### Statistical analysis

Statistical analysis was performed using GraphPad Prism v.10.4.1. Normality and lognormality tests were performed for each data set. Differences between two groups were analyzed using unpaired two-tailed Student’s *t*-tests. Statistical significance was indicated for *P < 0.05, **P < 0.01, and ***P < 0.001.

## Data Availability

Tn-seq data are deposited at the National Omics Data Encyclopedia (https://www.biosino.org/node) and are publicly available under accession number OEP00006176.

## SUPPLEMENTAL MATERIAL

Table S1 spectrum.01096-25-s0001.xlsx Tn insertion sites of the mutation library.

Table S2 spectrum.01096-25-s0002.xlsx Gene essentiality.

Table S3 spectrum.01096-25-s0003.xlsx Genes required for adaptation to stationary phase growth.

Table S4 spectrum.01096-25-s0004.xlsx Mutants with altered fitness upon drug exposure.

ASM does not own the copyrights to Supplemental Material that may be linked to, or accessed through, an article. The authors have granted ASM a non-exclusive, world-wide license to publish the Supplemental Material files. Please contact the corresponding author directly for reuse.

## References

[1] Organization WH. 2024. Global Tuberculosis Report 2024. Geneva

[2] Horsburgh CR, Barry CE III, Lange C. 2015. Treatment of tuberculosis. N Engl J Med 373:2149–2160. doi:10.1056/NEJMra1413919 26605929

[3] Mitchison DA. 1985. The action of antituberculosis drugs in short-course chemotherapy. Tubercle 66:219–225. doi:10.1016/0041-3879(85)90040-6 3931319

[4] Mitchison D, Davies G. 2012. The chemotherapy of tuberculosis: past, present and future. Int J Tuberc Lung Dis 16:724–732. doi:10.5588/ijtld.12.0083 22613684 PMC3736084

[5] Manina G, Dhar N, McKinney JD. 2015. Stress and host immunity amplify Mycobacterium tuberculosis phenotypic heterogeneity and induce nongrowing metabolically active forms. Cell Host Microbe 17:32–46. doi:10.1016/j.chom.2014.11.016 25543231

[6] Liu Y, Tan S, Huang L, Abramovitch RB, Rohde KH, Zimmerman MD, Chen C, Dartois V, VanderVen BC, Russell DG. 2016. Immune activation of the host cell induces drug tolerance in Mycobacterium tuberculosis both in vitro and in vivo. J Exp Med 213:809–825. doi:10.1084/jem.20151248 27114608 PMC4854729

[7] Paramasivan CN, Sulochana S, Kubendiran G, Venkatesan P, Mitchison DA. 2005. Bactericidal action of gatifloxacin, rifampin, and isoniazid on logarithmic- and stationary-phase cultures of Mycobacterium tuberculosis. Antimicrob Agents Chemother 49:627–631. doi:10.1128/AAC.49.2.627-631.2005 15673743 PMC547312

[8] Gengenbacher M, Rao SPS, Pethe K, Dick T. 2010. Nutrient-starved, non-replicating Mycobacterium tuberculosis requires respiration, ATP synthase and isocitrate lyase for maintenance of ATP homeostasis and viability. Microbiology (Reading) 156:81–87. doi:10.1099/mic.0.033084-0 19797356

[9] Rifat D, Bishai WR, Karakousis PC. 2009. Phosphate depletion: a novel trigger for Mycobacterium tuberculosis persistence. J Infect Dis 200:1126–1135. doi:10.1086/605700 19686042

[10] Rao SPS, Alonso S, Rand L, Dick T, Pethe K. 2008. The protonmotive force is required for maintaining ATP homeostasis and viability of hypoxic, nonreplicating Mycobacterium tuberculosis. Proc Natl Acad Sci USA 105:11945–11950. doi:10.1073/pnas.0711697105 18697942 PMC2575262

[11] Grant SS, Kaufmann BB, Chand NS, Haseley N, Hung DT. 2012. Eradication of bacterial persisters with antibiotic-generated hydroxyl radicals. Proc Natl Acad Sci USA 109:12147–12152. doi:10.1073/pnas.1203735109 22778419 PMC3409745

[12] Baek SH, Li AH, Sassetti CM. 2011. Metabolic regulation of mycobacterial growth and antibiotic sensitivity. PLoS Biol 9:e1001065. doi:10.1371/journal.pbio.1001065 21629732 PMC3101192

[13] Mishra R, Kohli S, Malhotra N, Bandyopadhyay P, Mehta M, Munshi M, Adiga V, Ahuja VK, Shandil RK, Rajmani RS, Seshasayee ASN, Singh A. 2019. Targeting redox heterogeneity to counteract drug tolerance in replicating Mycobacterium tuberculosis Sci Transl Med 11:eaaw6635. doi:10.1126/scitranslmed.aaw6635 31723039 PMC7212044

[14] Fan XY, Tang BK, Xu YY, Han AX, Shi KX, Wu YK, Ye Y, Wei ML, Niu C, Wong KW, Zhao GP, Lyu LD. 2018. Oxidation of dCTP contributes to antibiotic lethality in stationary-phase mycobacteria. Proc Natl Acad Sci USA 115:2210–2215. doi:10.1073/pnas.1719627115 29382762 PMC5834715

[15] Stanley S, Spaulding CN, Liu Q, Chase MR, Ha DTM, Thai PVK, Lan NH, Thu DDA, Quang NL, Brown J, Hicks ND, Wang X, Marin M, Howard NC, Vickers AJ, Karpinski WM, Chao MC, Farhat MR, Caws M, Dunstan SJ, Thuong NTT, Fortune SM. 2024. Identification of bacterial determinants of tuberculosis infection and treatment outcomes: a phenogenomic analysis of clinical strains. Lancet Microbe 5:e570–e580. doi:10.1016/S2666-5247(24)00022-3 38734030 PMC11229950

[16] Safi H, Gopal P, Lingaraju S, Ma S, Levine C, Dartois V, Yee M, Li L, Blanc L, Ho Liang HP, Husain S, Hoque M, Soteropoulos P, Rustad T, Sherman DR, Dick T, Alland D. 2019. Phase variation in Mycobacterium tuberculosis glpK produces transiently heritable drug tolerance. Proc Natl Acad Sci USA 116:19665–19674. doi:10.1073/pnas.1907631116 31488707 PMC6765255

[17] Bellerose MM, Baek SH, Huang CC, Moss CE, Koh EI, Proulx MK, Smith CM, Baker RE, Lee JS, Eum S, Shin SJ, Cho SN, Murray M, Sassetti CM. 2019. Common variants in the glycerol kinase gene reduce tuberculosis drug efficacy. MBio 10:e00663-19. doi:10.1128/mBio.00663-19 31363023 PMC6667613

[18] Brown MR, Collier PJ, Gilbert P. 1990. Influence of growth rate on susceptibility to antimicrobial agents: modification of the cell envelope and batch and continuous culture studies. Antimicrob Agents Chemother 34:1623–1628. doi:10.1128/AAC.34.9.1623 2285273 PMC171894

[19] Nandakumar M, Nathan C, Rhee KY. 2014. Isocitrate lyase mediates broad antibiotic tolerance in Mycobacterium tuberculosis. Nat Commun 5:4306. doi:10.1038/ncomms5306 24978671

[20] Eoh H, Rhee KY. 2013. Multifunctional essentiality of succinate metabolism in adaptation to hypoxia in Mycobacterium tuberculosis. Proc Natl Acad Sci USA 110:6554–6559. doi:10.1073/pnas.1219375110 23576728 PMC3631649

[21] Dutta NK, Klinkenberg LG, Vazquez MJ, Segura-Carro D, Colmenarejo G, Ramon F, Rodriguez-Miquel B, Mata-Cantero L, Porras-De Francisco E, Chuang YM, Rubin H, Lee JJ, Eoh H, Bader JS, Perez-Herran E, Mendoza-Losana A, Karakousis PC. 2019. Inhibiting the stringent response blocks Mycobacterium tuberculosis entry into quiescence and reduces persistence. Sci Adv 5:eaav2104. doi:10.1126/sciadv.aav2104 30906866 PMC6426458

[22] Lopatkin AJ, Stokes JM, Zheng EJ, Yang JH, Takahashi MK, You L, Collins JJ. 2019. Bacterial metabolic state more accurately predicts antibiotic lethality than growth rate. Nat Microbiol 4:2109–2117. doi:10.1038/s41564-019-0536-0 31451773 PMC6879803

[23] Bergkessel M, Basta DW, Newman DK. 2016. The physiology of growth arrest: uniting molecular and environmental microbiology. Nat Rev Microbiol 14:549–562. doi:10.1038/nrmicro.2016.107 27510862 PMC10069271

[24] Griffin JE, Gawronski JD, Dejesus MA, Ioerger TR, Akerley BJ, Sassetti CM. 2011. High-resolution phenotypic profiling defines genes essential for mycobacterial growth and cholesterol catabolism. PLoS Pathog 7:e1002251. doi:10.1371/journal.ppat.1002251 21980284 PMC3182942

[25] Long JE, DeJesus M, Ward D, Baker RE, Ioerger T, Sassetti CM. 2015. Identifying essential genes in Mycobacterium tuberculosis by global phenotypic profiling. Methods Mol Biol 1279:79–95. doi:10.1007/978-1-4939-2398-4_6 25636614

[26] DeJesus MA, Gerrick ER, Xu W, Park SW, Long JE, Boutte CC, Rubin EJ, Schnappinger D, Ehrt S, Fortune SM, Sassetti CM, Ioerger TR. 2017. Comprehensive essentiality analysis of the Mycobacterium tuberculosis genome via saturating transposon mutagenesis. MBio 8:e02133-16. doi:10.1128/mBio.02133-16 28096490 PMC5241402

[27] Vandal OH, Roberts JA, Odaira T, Schnappinger D, Nathan CF, Ehrt S. 2009. Acid-susceptible mutants of Mycobacterium tuberculosis share hypersusceptibility to cell wall and oxidative stress and to the host environment. J Bacteriol 191:625–631. doi:10.1128/JB.00932-08 19011036 PMC2620805

[28] Smeulders MJ, Keer J, Speight RA, Williams HD. 1999. Adaptation of Mycobacterium smegmatis to stationary phase. J Bacteriol 181:270–283. doi:10.1128/JB.181.1.270-283.1999 9864340 PMC103559

[29] Belisle JT, Vissa VD, Sievert T, Takayama K, Brennan PJ, Besra GS. 1997. Role of the major antigen of Mycobacterium tuberculosis in cell wall biogenesis. Science 276:1420–1422. doi:10.1126/science.276.5317.1420 9162010

[30] Armitige LY, Jagannath C, Wanger AR, Norris SJ. 2000. Disruption of the genes encoding antigen 85A and antigen 85B of Mycobacterium tuberculosis H37Rv: effect on growth in culture and in macrophages. Infect Immun 68:767–778. doi:10.1128/IAI.68.2.767-778.2000 10639445 PMC97204

[31] Ng VH, Cox JS, Sousa AO, MacMicking JD, McKinney JD. 2004. Role of KatG catalase-peroxidase in mycobacterial pathogenesis: countering the phagocyte oxidative burst. Mol Microbiol 52:1291–1302. doi:10.1111/j.1365-2958.2004.04078.x 15165233

[32] Park J, Cheon YJ, Jeong YC, Lee KS. 2024. Preliminary X-ray diffraction and ligand-binding analyses of the N-terminal domain of hypothetical protein Rv1421 from Mycobacterium tuberculosis H37Rv. Acta Crystallogr F Struct Biol Commun 80:135–141. doi:10.1107/S2053230X24005831 38935514 PMC11229554

[33] Rittershaus ESC, Baek S-H, Krieger IV, Nelson SJ, Cheng Y-S, Nambi S, Baker RE, Leszyk JD, Shaffer SA, Sacchettini JC, Sassetti CM. 2018. A lysine acetyltransferase contributes to the metabolic adaptation to hypoxia in Mycobacterium tuberculosis. Cell Chem Biol 25:1495–1505. doi:10.1016/j.chembiol.2018.09.009 30318462 PMC6309504

[34] Kanji A, Hasan R, Ali A, Zaver A, Zhang Y, Imtiaz K, Shi W, Clark TG, McNerney R, Phelan J, Rao S, Shafiq S, Hasan Z. 2017. Single nucleotide polymorphisms in efflux pumps genes in extensively drug resistant Mycobacterium tuberculosis isolates from Pakistan. Tuberculosis (Edinb) 107:20–30. doi:10.1016/j.tube.2017.07.012 29050768

[35] Walker TM, Miotto P, Köser CU, Fowler PW, Knaggs J, Iqbal Z, Hunt M, Chindelevitch L, Farhat MR, Cirillo DM, et al.. 2022. The 2021 WHO catalogue of Mycobacterium tuberculosis complex mutations associated with drug resistance: a genotypic analysis. The Lancet Microbe 3:e265–e273. doi:10.1016/S2666-5247(21)00301-3 35373160 PMC7612554

[36] Bhatt K, Banerjee SK, Chakraborti PK. 2000. Evidence that phosphate specific transporter is amplified in a fluoroquinolone resistant Mycobacterium smegmatis. Eur J Biochem 267:4028–4032. doi:10.1046/j.1432-1327.2000.01437.x 10866802

[37] Martini MC, Hicks ND, Xiao J, Alonso MN, Barbier T, Sixsmith J, Fortune SM, Shell SS. 2022. Loss of RNase J leads to multi-drug tolerance and accumulation of highly structured mRNA fragments in Mycobacterium tuberculosis. PLoS Pathog 18:e1010705. doi:10.1371/journal.ppat.1010705 35830479 PMC9312406

[38] Liu Q, Zhu J, Dulberger CL, Stanley S, Wilson S, Chung ES, Wang X, Culviner P, Liu YJ, Hicks ND, Babunovic GH, Giffen SR, Aldridge BB, Garner EC, Rubin EJ, Chao MC, Fortune SM. 2022. Tuberculosis treatment failure associated with evolution of antibiotic resilience. Science 378:1111–1118. doi:10.1126/science.abq2787 36480634 PMC9968493

[39] Nazarova EV, Montague CR, Huang L, La T, Russell D, VanderVen BC. 2019. The genetic requirements of fatty acid import by Mycobacterium tuberculosis within macrophages. Elife 8:e43621. doi:10.7554/eLife.43621 30735132 PMC6368401

[40] Bellerose MM, Proulx MK, Smith CM, Baker RE, Ioerger TR, Sassetti CM. 2020. Distinct bacterial pathways influence the efficacy of antibiotics against Mycobacterium tuberculosis. mSystems 5:mSystems doi:10.1128/mSystems.00396-20 PMC7406225 32753506

[41] Pandey AK, Sassetti CM. 2008. Mycobacterial persistence requires the utilization of host cholesterol. Proc Natl Acad Sci USA 105:4376–4380. doi:10.1073/pnas.0711159105 18334639 PMC2393810

[42] Tischler AD, Leistikow RL, Kirksey MA, Voskuil MI, McKinney JD. 2013. Mycobacterium tuberculosis requires phosphate-responsive gene regulation to resist host immunity. Infect Immun 81:317–328. doi:10.1128/IAI.01136-12 23132496 PMC3536151

[43] Rouse DA, DeVito JA, Li Z, Byer H, Morris SL. 1996. Site-directed mutagenesis of the katG gene of Mycobacterium tuberculosis: effects on catalase-peroxidase activities and isoniazid resistance. Mol Microbiol 22:583–592. doi:10.1046/j.1365-2958.1996.00133.x 8939440

[44] Manson AL, Cohen KA, Abeel T, Desjardins CA, Armstrong DT, Barry CE III, Brand J, Chapman SB, Cho S-N, Gabrielian A, et al.. 2017. Genomic analysis of globally diverse Mycobacterium tuberculosis strains provides insights into the emergence and spread of multidrug resistance. Nat Genet 49:395–402. doi:10.1038/ng.3767 28092681 PMC5402762

[45] Dowhan W. 2013. A retrospective: use of Escherichia coli as a vehicle to study phospholipid synthesis and function. Biochim Biophys Acta 1831:471–494. doi:10.1016/j.bbalip.2012.08.007 22925633 PMC3513495

[46] Tan Z, Khakbaz P, Chen Y, Lombardo J, Yoon JM, Shanks JV, Klauda JB, Jarboe LR. 2017. Engineering Escherichia coli membrane phospholipid head distribution improves tolerance and production of biorenewables. Metab Eng 44:1–12. doi:10.1016/j.ymben.2017.08.006 28867349

[47] Deng MZ, Liu Q, Cui SJ, Wang YX, Zhu G, Fu H, Gan M, Xu YY, Cai X, Wang S, Sha W, Zhao GP, Fortune SM, Lyu LD. 2024. An additional proofreader contributes to DNA replication fidelity in mycobacteria. Proc Natl Acad Sci USA 121:e2322938121. doi:10.1073/pnas.2322938121 39141351 PMC11348249

[48] Klepp LI, Sabio Y Garcia J, FabianaBigi. 2022. Mycobacterial MCE proteins as transporters that control lipid homeostasis of the cell wall. Tuberculosis (Edinb) 132:102162. doi:10.1016/j.tube.2021.102162 34952299

[49] Klepp LI, Forrellad MA, Osella AV, Blanco FC, Stella EJ, Bianco MV, Santangelo M de la P, Sassetti C, Jackson M, Cataldi AA, Bigi F, Morbidoni HR. 2012. Impact of the deletion of the six mce operons in Mycobacterium smegmatis. Microbes Infect 14:590–599. doi:10.1016/j.micinf.2012.01.007 22353253 PMC3615541

[50] Chen J, Fruhauf A, Fan C, Ponce J, Ueberheide B, Bhabha G, Ekiert DC. 2023. Structure of an endogenous mycobacterial MCE lipid transporter. Nature New Biol 620:445–452. doi:10.1038/s41586-023-06366-0 PMC12817965 37495693

[51] Alonso MN, Malaga W, Mc Neil M, Jackson M, Romano MI, Guilhot C, Santangelo MP. 2020. Efficient method for targeted gene disruption by homologous recombination in Mycobacterium avium subspecie paratuberculosis. Res Microbiol 171:203–210. doi:10.1016/j.resmic.2020.04.001 32283218

[52] Santangelo MP, Heuberger A, Blanco F, Forrellad M, Taibo C, Klepp L, Sabio García J, Nikel PI, Jackson M, Bigi F. 2016. Metabolic profile of Mycobacterium smegmatis reveals Mce4 proteins are relevant for cell wall lipid homeostasis. Metabolomics (Los Angel) 12:97. doi:10.1007/s11306-016-1035-4

[53] Helaine S, Conlon BP, Davis KM, Russell DG. 2024. Host stress drives tolerance and persistence: The bane of anti-microbial therapeutics. Cell Host Microbe 32:852–862. doi:10.1016/j.chom.2024.04.019 38870901 PMC11446042

[54] Dartois VA, Mizrahi V, Savic RM, Silverman JA, Hermann D, Barry CE. 2025. Strategies for shortening tuberculosis therapy. Nat Med 31:1765–1775. doi:10.1038/s41591-025-03742-3 40514466 PMC12278330

[55] DeMaio J, Zhang Y, Ko C, Young DB, Bishai WR. 1996. A stationary-phase stress-response sigma factor from Mycobacterium tuberculosis. Proc Natl Acad Sci USA 93:2790–2794. doi:10.1073/pnas.93.7.2790 8610119 PMC39711

[56] Darwin KH, Ehrt S, Gutierrez-Ramos JC, Weich N, Nathan CF. 2003. The proteasome of Mycobacterium tuberculosis is required for resistance to nitric oxide. Science 302:1963–1966. doi:10.1126/science.1091176 14671303

[57] Dong W, Nie X, Zhu H, Liu Q, Shi K, You L, Zhang Y, Fan H, Yan B, Niu C, Lyu LD, Zhao GP, Yang C. 2021. Mycobacterial fatty acid catabolism is repressed by FdmR to sustain lipogenesis and virulence. Proc Natl Acad Sci USA 118:e2019305118. doi:10.1073/pnas.2019305118 33853942 PMC8072231

[58] Yang M, Zhang L, Tao HL, Sun YC, Lou ZZ, Jia WZ, Hu LH, Gao CH. 2019. OxiR specifically responds to isoniazid and regulates isoniazid susceptibility in mycobacteria. FEMS Microbiol Lett 366:fnz109. doi:10.1093/femsle/fnz109 31125044

[59] Kusuya Y, Kurokawa K, Ishikawa S, Ogasawara N, Oshima T. 2011. Transcription factor GreA contributes to resolving promoter-proximal pausing of RNA polymerase in Bacillus subtilis cells. J Bacteriol 193:3090–3099. doi:10.1128/JB.00086-11 21515770 PMC3133182

[60] Nazarova EV, Montague CR, La T, Wilburn KM, Sukumar N, Lee W, Caldwell S, Russell DG, VanderVen BC. 2017. Rv3723/LucA coordinates fatty acid and cholesterol uptake in Mycobacterium tuberculosis Elife 6:e26969. doi:10.7554/eLife.26969 28708968 PMC5487216

[61] Wang X, Jowsey WJ, Cheung CY, Smart CJ, Klaus HR, Seeto NE, Waller NJ, Chrisp MT, Peterson AL, Ofori-Anyinam B, Strong E, Nijagal B, West NP, Yang JH, Fineran PC, Cook GM, Jackson SA, McNeil MB. 2024. Whole genome CRISPRi screening identifies druggable vulnerabilities in an isoniazid resistant strain of Mycobacterium tuberculosis. Nat Commun 15:9791. doi:10.1038/s41467-024-54072-w 39537607 PMC11560980

[62] Ofori-Anyinam B, Hamblin M, Coldren ML, Li B, Mereddy G, Shaikh M, Shah A, Grady C, Ranu N, Lu S, Blainey PC, Ma S, Collins JJ, Yang JH. 2024. Catalase activity deficiency sensitizes multidrug-resistant Mycobacterium tuberculosis to the ATP synthase inhibitor bedaquiline. Nat Commun 15:9792. doi:10.1038/s41467-024-53933-8 39537610 PMC11561320

[63] Waller NJE, Cheung CY, Cook GM, McNeil MB. 2023. The evolution of antibiotic resistance is associated with collateral drug phenotypes in Mycobacterium tuberculosis. Nat Commun 14:1517. doi:10.1038/s41467-023-37184-7 36934122 PMC10024696

[64] Bardarov S, Bardarov S, Pavelka MS, Sambandamurthy V, Larsen M, Tufariello J, Chan J, Hatfull G, Jacobs WR. 2002. Specialized transduction: an efficient method for generating marked and unmarked targeted gene disruptions in Mycobacterium tuberculosis, M. bovis BCG and M. smegmatis. Microbiology (Reading) 148:3007–3017. doi:10.1099/00221287-148-10-3007 12368434

[65] Perkowski EF, Miller BK, McCann JR, Sullivan JT, Malik S, Allen IC, Godfrey V, Hayden ID, Braunstein M. 2016. An orphaned Mce-associated membrane protein of Mycobacterium tuberculosis is a virulence factor that stabilizes Mce transporters. Mol Microbiol 100:90–107. doi:10.1111/mmi.13303 26712165 PMC5028898

[66] Santucci P, Johansen MD, Point V, Poncin I, Viljoen A, Cavalier JF, Kremer L, Canaan S. 2019. Nitrogen deprivation induces triacylglycerol accumulation, drug tolerance and hypervirulence in mycobacteria. Sci Rep 9:8667. doi:10.1038/s41598-019-45164-5 31209261 PMC6572852

